# *Crumbotti* and rose petals in a ghost mountain valley: foraging, landscape, and their transformations in the upper Borbera Valley, NW Italy

**DOI:** 10.1186/s13002-022-00535-7

**Published:** 2022-06-01

**Authors:** Michele F. Fontefrancesco, Dauro M. Zocchi, Roberta Cevasco, Rebekka Dossche, Syed Abidullah, Andrea Pieroni

**Affiliations:** 1 University of Gastronomic Sciences, Piazza Vittorio Emanuele 9, 12060 Bra/Pollenzo, Italy; 2 Department of Anthropology, Durham University, Durham, DH1 3LE UK; 3 Department of Antiquity, Philosophy, and History, University of Genoa, Via Balbi 2-6, 16126 Genoa, Italy; 4 Department of Botany, Abdul Wali Khan University Khyber Pakhtunkhwa, Mardan, 23200 Pakistan; 5 Department of Medical Analysis, Tishk International University, Qazi Muhammad Erbil, Kurdistan, 44001 Iraq

**Keywords:** Environmental anthropology, Ethnobotany, Ethnoecology, Landscape change, Historical ecology, Mountain development

## Abstract

**Background:**

The abandonment of mountain areas in Europe is a process that started during industrialisation and whose traces are still present nowadays. Initiatives aimed at stopping this decline and preserving the local biological and cultural diversities reflect the crucial issue of fostering sustainable rural development. This article contributes to the ongoing debate in assessing and preserving local ecological knowledge (LEK) in a highly marginalised mountain community in the Piedmontese Apennines to support local development. In so doing, it continues a larger project assessing how local botanical knowledge and landscapes evolve over time, in order to understand in more depth which factors affect how LEK is shaped, eroded, and re-created, and how this could be revitalised.

**Methods:**

We compared information about the current gathering and use of local wild plants in the upper Borbera Valley (Carrega Ligure municipality, NW Italy), elicited via 34 in-depth open and semi-structured interviews, with the findings of a field study conducted in the same location, most likely carried out at the end of the 1970s and published in 1981.

**Results:**

There were remarkable quantitative and qualitative differences between the two ethnobotanies. The gathering and use of some wild medicinal plants growing in meadows, woodlands, and higher mountain environments (*Achillea*, *Centaurea*, *Dianthus*, *Ostrya*, *Picea*, *Polygonum*, *Potentilla*, and *Thymus*) seems to have disappeared, whereas the collection of plants growing in more anthropogenic environments, or possibly promoted via contacts with the “reference” city of Genoa (the largest city close to Carrega and historically the economic and cultural centre to which the valley was mostly connected), has been introduced (i.e. ramsons, safflower, bitter oranges, black trumpets) or reinvigorated (rose petals). This trend corresponds to the remarkable changes in the local landscape ecology and agro-silvo-pastoral system that took place from the first half of the twentieth century, dramatically increasing woodland and secondary vegetation, and decreasing coppices, plantations, grasslands and segregating cultivated land.

**Conclusion:**

The findings show a very difficult rearrangement of the LEK, as most of the areas the local actors still know are within their villages, and they no longer have daily experience in the rest of the abandoned woodland landscape (except for mushrooming and gathering chestnuts). This situation can be interpreted in two ways: as the start of the complete abandonment of the valley, or as a starting residual resilience lynchpin, which could possibly inspire new residents if the larger political-economic framework would promote measures for making the survival of the mountain settlements of this municipality possible, and not just a chimera.

## Background

Rural abandonment has been at the centre of public debate in recent years. The FAO [1] has indicated this abandonment, and the consequential urbanisation, as one of the main challenges for the future of the world’s food system in terms of resilience and sustainability. Rural abandonment is occurring internationally, in both developed and developing countries [2, 3]. When the phenomenon is not caused by famine, persecution or war [4, 5], it is directly linked to an individual’s search for prosperity, moved by what Ferguson [6] inspiringly defined as the “expectation of modernity”. In this respect, there is a cultural and social continuity between the present events and the migratory phenomena that occurred during the twentieth century, which changed the geography of Europe, especially in mountain areas [7–9]. Migration is thus a key force in terms of the transformation of a community’s relationship with its surroundings, as well as with its local ecological knowledge (LEK) [10–13]: a change that cannot be described only in terms of opposition between tradition and modernity or between rural and urban [14], and not only in terms of sociocultural erosion [15]. In so far as LEK is situated knowledge [16], the analysis of rural migration raises questions concerning the spatial reorientation and cultural adaptation of local communities.

The work of Lauer and Aswani [17] kindled a debate about the situatedness of LEK. Their research on the Western Salomon Islands showed the link between cognitive aspects and other modalities of knowing, and how it is deeply intertwined with everyday ecological practices, which encompass activities such as farming, managing woodlands, foraging and herding. Since then, other scholars [18–20] have highlighted the close relationship between LEK and the ways in which the environment is lived by a community, so that LEK appears to be an expression of the “dwelling perspective” [21], and the embeddedness of human beings in the world. LEK is thus intrinsically dynamic [22] and responds to and derives from the ways in which a community looks at its surroundings and the resources available therein. It is affected by the changes undergone by other communities and the vast world, often in an unsymmetrical way [23]. Various factors, such as government policies, modern science and technology, education, and market development, can operate [24, 25]. Migration is one of them [26].

While recent decades have witnessed a deep transformation of LEK in rural communities, in order to understand how LEK can be sustainably promoted as a potential resource for the reactivation of marginalised localities and communities [27], it is crucial to critically explore how specific dynamics have driven their evolution and conservation, as well as how specific phenomena have shaped the perception of community members.

To this end, particular attention should be paid to the continuities and changes of LEK across time and space. In fact, rural abandonment cannot be described a priori as an erosion of LEK, but rather a qualitative and quantitative change that affects the community and expresses its social and economic transformations [28]. A diachronic comparison would thus help in shedding light on the complex transformations underpinning this phenomenon. Changes in LEK assessed using comparable research methods (ethnography-based techniques, i.e. face-to-face interviews) remain limited, however, due to the lack of reliable ethnobotanical studies conducted in previous decades [28–32].

An interesting opportunity for diachronic analysis is provided by one study that was conducted between 1976 and 1978 in Carrega Ligure, a village in the Piedmontese Apennines in Italy, in which an Italian researcher documented folk herbal practices and the botanical taxa used by the community via face-to-face interviews with locals [33]. The location of the survey is an example of a peripheral rural area in Northern Italy that endured dramatic emigration during the second half of the twentieth century, reducing the local population from over 2,000 inhabitants in 1921 to less than 90 in 2021, according to the periodic national census. We decided to conduct the current field research in exactly the same area, in order to analyse the ethnobotanical picture forty years later.

The present paper contributes to the aforementioned debate by exploring changes in the LEK of a mountain community over the past four decades, using a comparison between the plants gathered and used by the local community in the mid-seventies and those gathered and used in the year 2021 as a proxy for the deeper socio-economic change that the community endured, building on the results of previous historical research into the profound changes in the local ecology.

The specific objectives of this study were therefore:

- to investigate the current ethnobotanical knowledge (specifically focusing on both wild food and herbal plant uses) of Carrega;
- to compare currently recorded herbal uses with those recorded forty years earlier and to parallel that with a comparison of landscape transformations over the past century;
- to interpret possible differences in ecological and cultural terms, and to discuss the possible diachronic changes/transformations of LEK;
- to explore the current trajectories of traditional knowledge revitalisation and their possible development.

## Materials and methods

### Selection of the ethnobotanical literature

A decade ago, our research group started an investigation into the historical ethnobotanical literature of Italy from 1884 to the 1980s [34, 35] and selected those works which included both reliable botanical identifications (i.e. works indicating voucher specimens, or studies conducted by botanists) and some clear indications that the ethnobotanical data were possibly collected via face-to-face interviews with local community members. At the end of this months-long work, we had obtained no more than a dozen field studies conducted between 1955 and 1980 in NW, NE, and Central Italy, which could be considered appropriate for diachronic comparison. One study, conducted at the end of the 1970s in the Ligurian Apennines and published in 1981 [33], stood out among this restricted group. This field study was conducted by Enrico Martini, a botanist at the University of Genoa, via face-to-face interviews with locals, focusing on both wild and cultivated medicinal plants gathered and used in domestic herbal practices, and/or as medicinal beverages (e.g. teas, herbal liqueurs). This study is limited since it did not document wild food botanicals (e.g. wild vegetables and fruits) and mushrooms, local plant names, frequency of quotation, or use. The work also did not provide detailed information regarding the ecological areas in which the plants were collected. On the other hand, the work indicated the exact hamlets where the information was collected for each individual quoted plant.

### Study area

The Borbera Valley is mainly mountainous (max 1,700 m a.s.l.) with a predominantly marly clay/marly calcareous substrate. The most important mountain peaks are Mount Legnà (1,669 m a.s.l.), Mount Carmo (1,642 m a.s.l.), and the Antola Massif (1,597 m a.s.l.). The habitat is typical of the Apennine, with chestnut and beech tree woodlands dominating up to 1,000 m, while higher elevations (1,100–1,700 m a.s.l.) are covered by fir, pine, and birch forests, as well as grasslands [36].

In 2005, the Upper Valley was established as a Natura 2000 Site of Community Importance (IT1180011) within the European Habitats and Bird Directives. The area (approximately 5,500 hectares) was turned into a regional park in 2019 by the region of Piedmont, called Upper Borbera Valley Natural Park (Parco Naturale Alta Val Borbera).

**Fig. 1:**
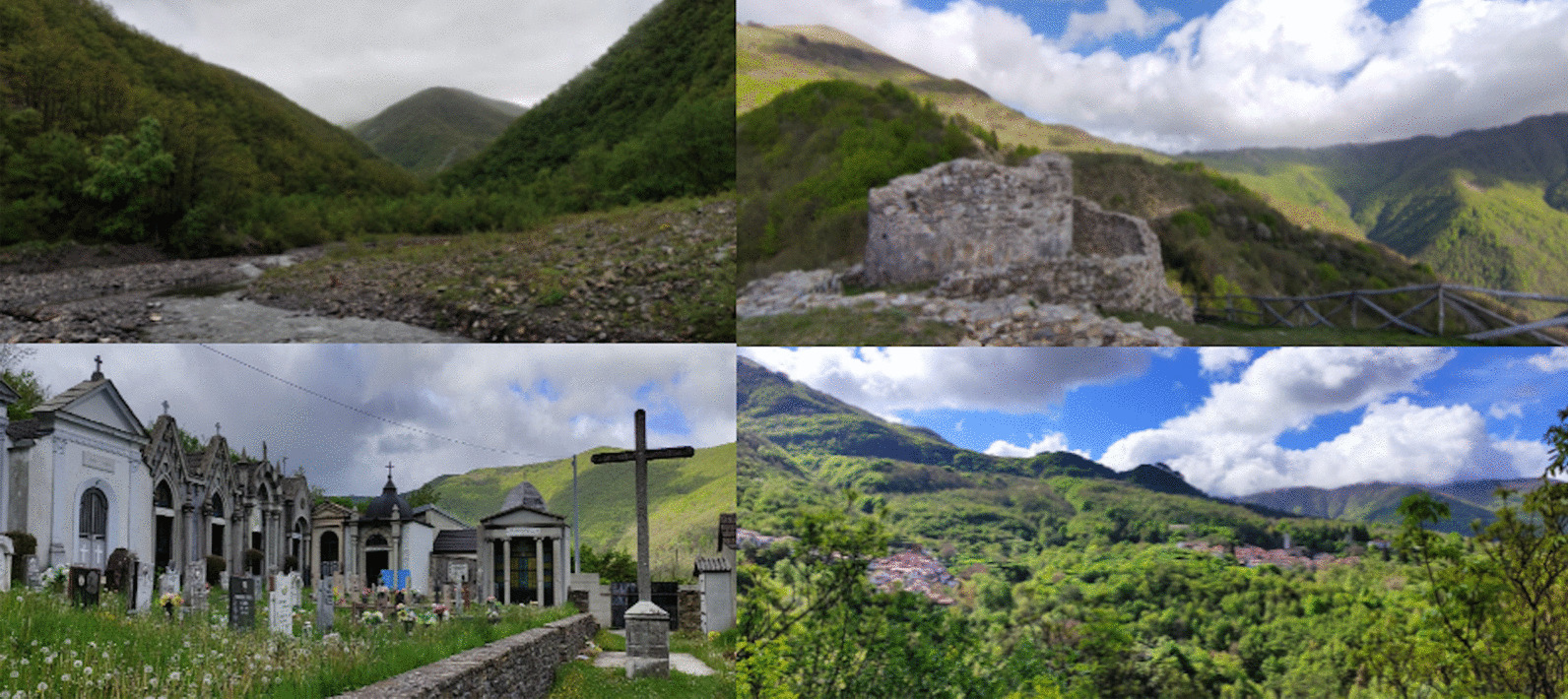
Current landscape of Carrega Ligure (Photograph: Michele Filippo Fontefrancesco)

The entire upper Borbera Valley is part of the territory of the municipality of Carrega Ligure (Fig. 1). The municipality lies in the south-eastern corner of Piedmont, about 70 km south-east Alessandria, right on the border with Liguria. Historically, the municipality was part of the territories of the division of Genoa, from which it was separated in 1859 and made part of the province of Alessandria. Despite this administrative change, the municipality is still heavily influenced by Genoa in terms of culture and economy. People in Carrega speak Italian and a local variant of Ligurian, a Gallo-Italic language spoken as the main language in Liguria and southern Piedmont until the second half of the twentieth century [37]. Moreover, they commonly commute to Genoa for work, higher education, and medical treatment.

On 1 January 2021, the municipality had 88 inhabitants—although fewer than 40 were permanent residents—who live in sparse and largely abandoned hamlets, some of which are only inhabited during the summer. The present demography is the result of a dramatic depopulation of the area that occurred between the late nineteenth century and the first half of the 20th. The village lies on a major medieval road that connected the Ligurian Sea with the Po Plain; however, its role declined after the construction of the communication road “Strada dei Giovi” in 1821 and opening of the Genoa–Turin railroad in the neighbouring Scrivia Valley in the 1850s. Economic marginalisation, as well as the opening of new industries in the main cities along the new railroad, led to demographic decline between 1861 and 1971 involving the loss of 91% of its population [37]. While people from Carrega largely moved abroad to the Americas in the nineteenth century, since the beginning of the twentieth century, migrants have mostly headed to Genoa, the largest city close to the village, and the towns in the nearby Scrivia Valley.

Depopulation led to a crisis in the traditional economic system based on agriculture (in 1929 91.2% of its territory was still occupied by agriculture or forestry, and in 1951 89.54% of the local population was still involved in this trade): it involved the cultivation of terraces with wheat, barley, and small-scale gardening; cattle breeding and pastoralism, which supported local dairy production and a consistent supply of livestock that were sold across Piedmont, Liguria and Lombardy; and hunting and gathering practices (including the collection of chestnuts, mushrooms, and wild herbs used both as food and medicine) mainly carried out in woodlands and higher pastures [37]. Particularly after the 1970s, the continuous depopulation resulted in the progressive erosion of commercial and public services offered in town: the local elementary school closed in the 1980s, and shops rapidly disappeared from the town, with the last bar-restaurant closing in 2020 during the COVID-19 pandemic. The contemporary economy of the town relies mostly on pensions and remittances, but the municipality has attempted to develop actions, including the establishment of the natural park, to develop a future sustainable strategy based on tourism activities (hiking, hunting, eco-gastronomy), environmental conservation initiatives and the reactivation of the local economy (wood and food production). However, tourism remains limited to the summer period (mostly linked to the return of people who still own houses in the municipality during summer holidays) and to autumn (Carrega is a destination for hunters, and mushroom and chestnut gatherers who come from Emilia-Romagna, Liguria, Lombardy, and Piedmont).

### Current field ethnobotanical study

**Fig. 2:**
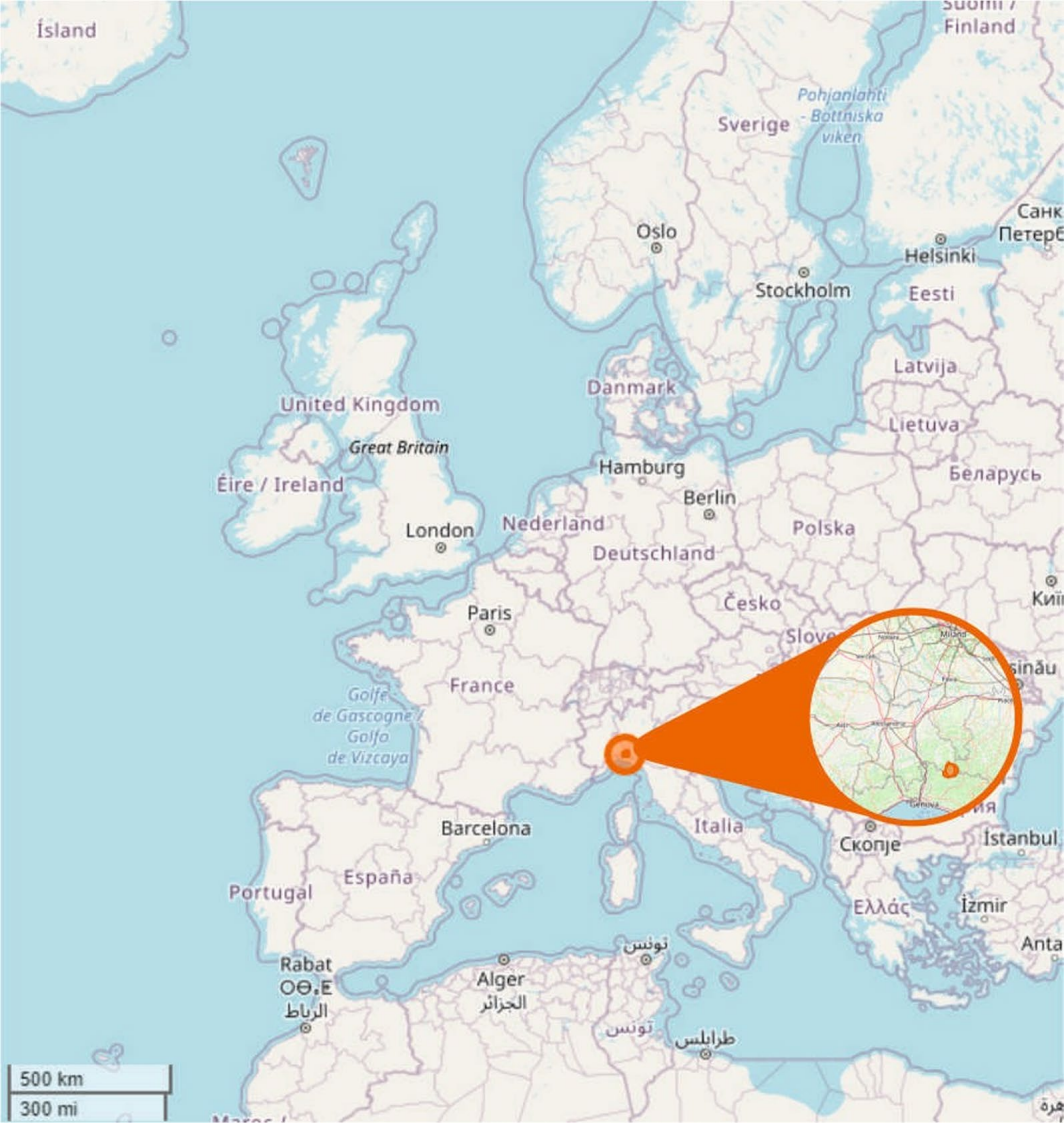
Map of the study area based on Openstreetmap cartography (Figure: Michele F. Fontefrancesco)

**Fig. 3:**
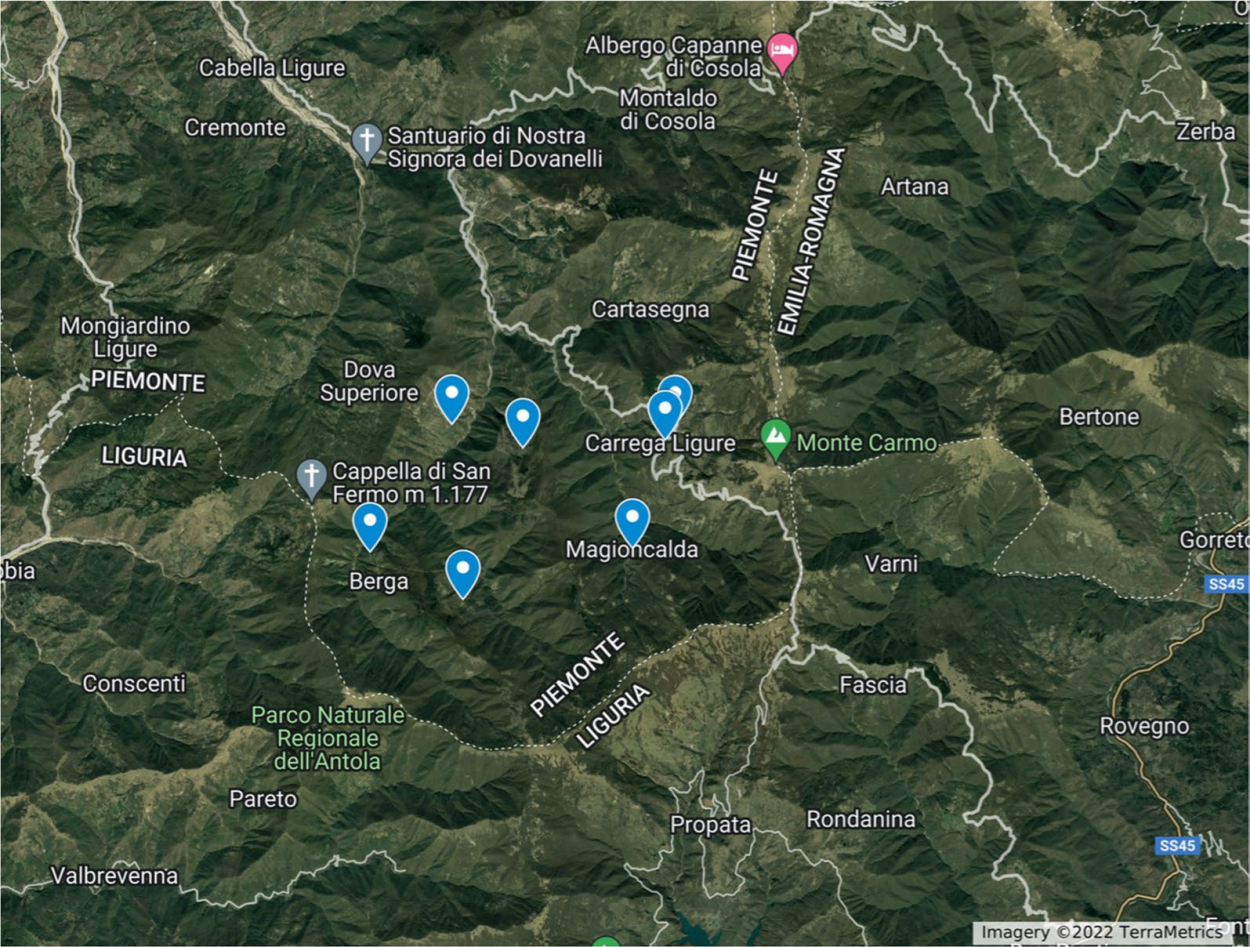
Map highlighting the hamlets visited within the municipality of Carrega Ligure: Agneto (44.62146996379504, 9.132656269739753), Berga (44.60256611969035, 9.115640313056668), Carrega (44.61910660260257, 9.17694869135217), Connio (44.621380782100445, 9.178953440279466), Croso (44.59554098130246, 9.134945790463432), Magioncalda (44.603140247072844, 9.170182663722551), and Vegni (44.61794718269442, 9.14744129307855) (File credits: Creative Commons Attribution-Share Alike 3.0 license)

The current field research was carried out in one of the municipalities where the 1970s study was conducted: Carrega Ligure (Fig. 2). The following map (Fig. 3) highlights the hamlets visited during the research.

The ethnobotanical field study was carried out by two authors (AP and MFF) between May and July 2021. In-depth open and semi-structured interviews were conducted with community members (n = 34, average age 65) in the aforementioned hamlets. Participants were selected using snowball sampling techniques, focusing specifically on those elderly community members (e.g. farmers, housewives) who still have connections to an agro-pastoral way of life. Informants were asked about current gathering and gathering in the recent past, and the use of: a) local wild and semi-domesticated food and medicinal plants; b) food mushrooms; and c) those few cultivated plants which could be considered “unusual” in local use (compared to the normal food uses of plants in Italy and in the macro-regional study area). Study participants were specifically asked about the local plant name(s) and plant part(s) used, as well as specific details about their manipulation/preparation and current herbal or food use(s). They were particularly asked to mention if the uses they quoted were current, had disappeared, or were “new” (i.e. newly introduced during their lifetime). Interviews were conducted in standard Italian. Informed consent was verbally obtained before the interviews, and researchers adhered to the ethical guidelines of the International Society of Ethnobiology [38]. Informants were always asked to show the quoted plants or, at least, to describe them during the interviews. Only three taxa (*Lactuca*, *Stachys*, and *Polygala*) had not been reported in neighbouring areas in previous studies, and these were identified by AP using the Flora of Italy [39]. Botanical nomenclature was later standardised using the Plant List Database [40], family assignments follow the Angiosperm Phylogeny Group IV system [41], and mushroom nomenclature follows the Index Fungorum database [42].

### Ethnobotanical data analysis

The ethnobotanical data recorded during the fieldwork were tabulated and compared with that arising from the medical-ethnobotanical study that was conducted at the end of the 1970s in the same area [33]. The recorded plant reports were also qualitatively compared with the most comprehensive worldwide wild food plant compendia [43, 44] and the Italian ethnobotanical database [45], in order to indicate possible novel reports (i.e. uses not previously recorded).

### Historical ecological analysis

The use and comparison of different sources, such as historical and recent cartographic and photographic material, cadastral maps, digital terrain models (DTM), archival documents and field surveys, have been shown to be very useful in obtaining a profound understanding of landscape changes through time. The analysis contained a time span of 200 years, starting with the Napoleonic land register (1811) in combination with historic maps from 1828 (Minute di Campagna, Corpo di Stato Maggiore, Fogli 88, 93, O.14) and 1853 (Gran Carta degli Stati Sardi di Terraferma (Fogli 62 and 68), and then topographic maps from 1877 (Fogli 71 SE and SO), 1902 (Foglio 83), 1935 (Fogli 71 SE and SO), 1937 (Foglio 83), and 1959 (Fogli 71 SE and SO). More recent sources included aerial photographs from 1936, 1954, 1981, and 2000 in combination with the DTM (10 m resolution), the recent land register of 1950, orthographic photographs from 2009 and 2011, and a Google Earth consultation in 2014.

Moreover, regression spatial analysis has proven to add value to the understanding of historic asynchronies and anomalies, instead of simplistic generalisations [46, 47]. Its related methodology was described by RD [48]. It focused on a GIS environment relating land covers with a series of attributes (diagnostic, differentiating, and descriptive) [49]. The combination of the level of abandonment with land cover, its function and use, hillslope, field structure, the presence of terraces and small landscape elements, yields a comprehensive overview of how and where abandonment started and how it has changed.

## Results

### Foraged plants and mushrooms for food and medicine in the upper Borbera Valley

Fifty-nine wild and semi-domesticated food and medicinal plants and mushrooms, including two unidentified taxa, were recorded in total. We also documented the use of nine cultivated plants that exhibited unusual local uses. Table 1 shows this remembered and current foraging food and ethnobotanical knowledge of the upper Borbera Valley, and Table 2 lists the wild herbals recorded four decades ago.

**Table 1:** Wild and semi-domesticated food plants and herbal remedies used in the upper Borbera Valley (the table also includes a few cultivated plants whose local use is peculiar)

Botanical taxon and family	English name	Local name(s)	Used part(s)	Local culinary or herbal preparation(s) and use(s)	Frequency of quotations
*Agaricus campestris* L., Agaricaceae	Field Mushroom	Balun, Masin, Prale (pl.)	Fruiting body	Fried, *risotto*, sauces	+ +
*Allium sativum* L., Amaryllidaceae C	Garlic	Agio	Bulbs	Necklaces worn by children as an anthelmintic; consumed as a hypotensive	+ +
*Allium ursinum* L., Amaryllidaceae	Ramsons	Aglio ursino	Leaves	Sauces	+ N
*Amanita caesarea* (Scop.) Pers., Amanitaceae	Caesar's mushroom	Ovulo	Fruiting body	Eaten raw or fried	+ +
*Apium nodiflorum* (L.) Lag., Apiaceae	Fool's watercress (marsh celery)	Cresciun	Aerial parts	Boiled (considered diuretic)	+ *
*Arnica montana* Hook., Compositae	Arnica	Arnica	Flowers	External compresses or oleolite or alcohol macerate, externally applied to treat rheumatism; tea	+ *
*Balsamita major* Desf., Asteraceae C	Costmary	Erba amaa	Leaves	Omelettes	+
*Boletus* spp., Boletaceae	Boletus	Funzo negro, Riueu, Verie (pl.)	Fruiting body	Fried, *risotto*, sauces	+ + +
*Borago officinalis* L., Boraginaceae	Borage	Buraje	Leaves	Omelettes, soups, boiled as filling for ravioli	+ +
*Cantharellus cibarius* Fr., Hydnaceae	Chanterelle	Galeti, Galette (pl.)	Fruiting body	Fried, sauces	+ + +
*Carthamus tinctorius* L., Compositae C	Safflower	Saffarano	Petals	Used to colour *risotto* yellow	+ N
*Castanea sativa* Mill., Fagaceae	Chestnut	Castagna	Fruits	Roasted or boiled; traditionally cooked with rice or potato dumplings; dried, boiled in milk; dried and powdered into flour to make *polenta*	+ + +
*Chelidonium majus* L., Papaveraceae	Greater celandine	Erba da calli	Latex	Externally applied on warts and burns	+ *
*Chenopodium bonus-henricus* L., Chenopodiaceae	Good-King-Henry	Spinasso	Aerial parts	Boiled and used as filling for ravioli	+
*Citrus x aurantium* L., Rutaceae	Bitter orange	Melangolo	Fruit peels	Macerated in new wine to obtain an aromatised wine	+ N
*Clematis vitalba* L., Ranunculaceae	Traveller's joy	Ligabosca, Viassu	Young shoots	Omelette	+ + +
*Cornus mas* L., Cornaceae	Cornelian cherry	Curnaghe (pl.), Curnà	Fruits	Eaten raw, jam	+ *
*Corylus avellana* L., Betulaceae	Hazelnut	Niusse, Nissue (pl.)	Kernels	Consumed raw or dried, oil*	+
*Crataegus* spp., Rosaceae	Hawthorn	Spina	Fruits	Eaten raw as a snack	+ *
*Craterellus cornucopioides* L., Hydnaceae	Black trumpet	Santacaten	Fruiting body	Fried, sauces	+ N
*Cynodon dactylon* (L.) *Pers.*, Poaceae	Bermuda grass	Gramigna	Whole plant	Tea	+ *
*Fragaria vesca* L., Rosaceae	Wild strawberry	Mulette, Meieli, Mieirui (pl.)	Fruits	Eaten raw, jam, syrup	+
*Gentiana ligustica* R.Vilm. & Chopinet, Gentianaceae	Narrowleaf gentian	Cancagè	Flowers	Macerated in white wine or alcohol as a stomachic; cold macerated in water as a digestive; tea as a digestive and mouth anti-inflammatory	+ +
*Gentiana lutea* Ruiz & Pav. ex G.Don, Gentianaceae	Gentian	Gensana, Reise de drago	Roots	Macerated in alcohol or white wine as a digestive	+ +
*Grifola frondosa* (Dicks.) Grey, Grifolaceae	Hen-of-the-woods	Barbegin	Fruiting body	Deep fried, *risotto*, boiled in vinegar and pickled in olive oil	+
*Juglans regia* L., Juglandaceae	Walnut	Noce	Kernels and unripe fruits	Kernels: dried, sauces, liqueur Unripe fruits: dye for hair	+ + *
*Lactuca perennis* L., Asteraceae	Wild lettuce	Crumbotti (pl.)	Young aerial parts	Salads, boiled, filling for ravioli	+ + +
*Laurus nobilis* L., Lauraceae	Bay leaf	Faggiu, Ofegiu	Leaves	Seasoning	+
*Macrolepiota procera* (Scop.) Singer, Agaricaceae	Parasol mushroom	Trulla	Upper fruiting body	Fried	+ +
*Malus domestica* Borkh., Rosaceae C	Apple	Puma	Seeds	Seeds: macerated in alcohol as a liqueur	+ *
*Malus sylvestris* (L.)Mill., Rosaceae	European crab apple	Puma sarvaega	Fruits	Fermented into home-made cider, vinegar	+ *
*Malva sylvestris* L., Malvaceae	Mallow	Varma	Whole plants (aerial parts and roots)	Decoction as a depurative, bechic, and mouth anti-inflammatory; put in hot water—fumigation as anti-cold	+ + +
*Matricaria recutita* L., Asteraceae	Chamomile	Camomilla	Flowering tops	Tea as a spasmolytic (especially against toothaches)	+
*Mentha spicata* L., Lamiaceae C	Spearmint	Menta	Leaves	Syrup	+
*Olea europaea* L., Oleaceae	Olive leaf	Uiva	Leaves	Tea, as a hypotensive	+ +
*Origanum vulgare* L., Lamiaceae	Wild oregano	Curnabugia	Flowering tops and aerial parts	Seasoning	+ +
*Polygala vulgaris* L., Polygalaceae	Milkwort	Poligala	Flowering tops	Tea as a bechic	+ *
*Primula vulgaris* Huds, Primulaceae	Primrose	Cucchi (pl.)	Leaves	Salads, soup (sometimes considered good for mitigating prostatitis)	+ + +
*Prunus avium* (L.) L., Rosaceae	Sweet cherry	Siege (pl.)	Fruits	Eaten raw, jam, syrup	+ +
*Prunus cerasus* L., Rosaceae (Diverse landraces) C	Wild cherry	Maine, Graffiun, Visciue (pl.)	Fruits	Eaten raw, jam, syrup	+ +
*Prunus spinosa* Walter, Rosaceae	Blackthorn	Brugnini (pl.)	Fruits	Macerated in alcohol to obtain a home-made liqueur	+ *
*Pyrus communis* L., Rosaceae (Local landraces)	Pear	Pera rosetta, Pera nissa	Fruits	Baked	+ + *
*Pyrus pyraster* (L.) Burgsd., Rosaceae	Wild pear	Pera saravega	Fruits	Fermented into a home-made cider	+ *
*Quercus* spp., Fagaceae	Oak	Seru	Bark	Externally applied to cuts (especially in folk veterinary medicine)	+ *
*Robinia pseudoacacia* L., Fabaceae	Black locust	Gaggia	Inflorescences	Deep fried	+ +
*Rosa canina* Siev., Rosaceae	Dog rose	Grattacül, Scaganissi	Pseudo fruits	Eaten raw as a snack, tea as a bechic and anti-diabetic; jams	+ +
*Rosa* spp., Rosaceae C	Rose	Röza	Petals	Syrup (sometimes also considered a mild laxative)	+ + +
*Rubus idaeus* L., Rosaceae	Raspberry	Lampöna, Ampöine	Fruits	Eaten raw, jams, syrup	+ +
*Rubus ulmifolius* Schott, Rosaceae	Blackberry	Muie (pl.)	Fruits and young shoots	Fruits: eaten raw, jam, syrup Young shoots: eaten raw as a snack	+ +
*Rumex acetosella* L., Polygonaceae	Sorrell	Erba brisca	Young aerial parts	Snack	+ *
*Russula cyanoxantha* (Schaeff.) Fr., Russulaceae	Charcoal burner	Pévègn, Crumbette	Fruiting body	Fried, sauces, boiled in vinegar and pickled in olive oil	+ +
*Russula vesca* Fr., Russulaceae	Bare-toothed russula	Sementin	Fruiting body	Fried or baked with potato slices	+
*Ruta graveolens* L., Rutaceae C	Rue	Erba rüa	Aerial parts	Liqueur	+
*Sambucus nigra* L., Adoxaceae	Elderberry	Sambügo	Flowers	Beverage prepared by fermenting for nine days with lemon, vinegar, sugar; syrup as a bechic	+ +
*Sedum* sp., Crassulaceae	Stonecrop		Leaves	Externally applied to treat skin problems	+ *
*Solanum tuberosum* L., Solanaceae C	Potato	Patata	Tubers	Sliced and externally applied on the forehead as a diaphoretic	+ *
*Sorbus aria* (L.) Crantz, Rosaceae	Whitebeam	Anigüe (pl.)	Fruits	Eaten raw as a snack	+ *
*Stachys annua* (L.) L., Lamiaceae	Annual hedge-nettle	Gerba	Leaves	Tea	+ + *
*Taraxacum officinale* (L.) Weber ex F.H.Wigg., Compositae	Dandelion	Dente de can	Leaves	Salads, soup, boiled to colour home-made noodles green	** + + **
*Tilia cordata* Mill., Malvaceae	Lime tree flower	Teie	Flowers	Tea	** + + **
*Trifolium* spp., Fabaceae	Clover	Trifoglio	Flowers	Sucked	+ *
*Urtica dioica* L., Urticaceae	Nettle	Begìa, Besciga, Urtiga	Leaves	Soup (often with onions and bacon or the so-called 12 apostles’ soup of Holy Friday, also made with dandelion and borage leaves, other cultivated vegetables and basil), omelettes	** + + + **
*Vaccinium myrtillus* L., Ericaceae	Blueberry	Scurnogiotti (pl.)	Fruits	Eaten raw, jam, syrup	** + **
*Vaccinium uliginosum* L., Ericaceae	Bog bilberry	Peiette (pl.)	Fruits	Eaten raw, jam, syrup	** + **
*Viola odorata* L., Violaceae	Violet	Violetta	Flowers	*Risotto*, liqueur	** + **
Diverse spp., Poaceae	Hay	Feno	Aerial parts	Put in hot water and fumigation as anti-cold	+ *
Unidentified taxon	-	Grigiuielu	Leaves	Omelettes	+ *
Unidentified taxon	-	Campanele	Aerial parts	Tea, blood depurative	+ *

C: cultivated plant; (pl.): folk name(s) expressed in plural; + + + : quoted by more than 40% of the study participants; +  + : quoted by 10–40% of the informants; + : quoted by one or two informants only; *: use reported only for the past; N: recently introduced “new” use

**Table 2:** Wild plant-based domestic remedies recorded in the Upper Borbera Valley approximately 45 years ago (in bold are those botanical genera matching the ones recorded during the current field study)

Botanical taxon and family	English name	Used part(s)	Herbal preparation(s) and use(s)
*Achillea millefolium* L., Asteraceae	Yarrow	Flowering tops	Tea as a sedative; externally applied as a vulnerary
*Anchusa azurea* Mill., Boraginaceae	Italian bugloss	Flowering tops	Decoction as a bechic
*Centaurea scabiosa* L., Asteraceae	Greater knapweed	Roots	Macerated in alcohol and drunk as a liver protector
*Centaurea uniflora* Turra, Asteraceae	Knapweed	Flowering tops	Tea as an anti-diarrheal, depurative and diuretic
*Dianthus carthusianorum* L., Caryophyllaceae	Carthusian pink	Petals	Wine macerate as an anti-neuralgic
***Mentha**** longifolia* (L.) L., Lamiaceae	Mint	Flowering tops	Tea as a stomachic
*Onobrychis viciifolia* Scop., Fabaceae	Sainfoin	Roots	Externally applied as a vulnerary
*Ostrya carpinifolia* Scop., Betulaceae	Hop hornbeam	Bark	Decoction as a diuretic and blood depurative; externally applied as an anti-eczema agent and an anti-haemorrhoidal
*Papaver rhoeas* L., Papaveraceae	Common poppy	Flowers	Tea as a sedative
*Picea abies* (L.) H.Karst., Pinaceae	Spruce	Shoots	Decoction as a bechic
*Plantago lanceolata* L., Plantaginaceae	Ribwort plantain	Whole plant	Tea as a stomachic; chewed leaves and roots against toothache
*Polygonum bistorta* L., Polygonaceae	Bistort	Rhizome	Tea against menstrual pains; in gargles as an antiseptic; externally applied as a vulnerary
*Potentilla reptans* L., Rosaceae	Cinquefoil	Whole plant	Decoction as an anti-diarrhoeal
***Rosa**** pendulina* L., Rosaceae	Mountain rose	Petals	Tea as an astringent and tonic
***Sedum**** anacampseros* L., Crassulaceae	Love restorer	Aerial parts	Compresses to treat wounds
*Thymus pulegioides* L., Lamiaceae	Wild thyme	Leaves	Externally applied as a vulnerary

**Fig. 4:**
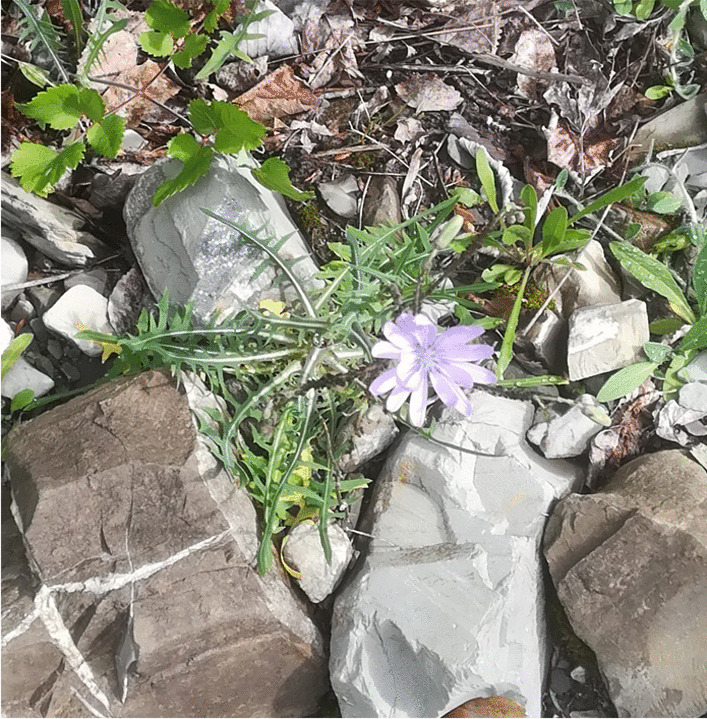
*Crumbotti* (*Lactuca perenni*s, photograph: Andrea Pieroni)

A few of the plants recorded in the current study were extensively quoted, i.e. by more than 40% of the study participants. Among the plants collected mainly for proper food (non-herbal) preparations, boletus (*Boletus* spp.), chestnuts (*Castanea sativa*), nettles (*Urtica dioica*), rose petals (*Rosa* spp., see following paragraph), and two other lesser-known wild plants emerged as most important within the food and ethnobotanical knowledge of the upper Borbera Valley: the shoots of traveller’s joy (*Clematis vitalba*), used for an iconic spring omelette (similar uses are and especially were known in rural Italy [45]), and the aerial parts of a wild lettuce (*Lactuca perennis*, locally known as *crumbotti*, Fig. 4), which we may consider the real plant cultural marker of the area.

Wild *Lactuca* species are, in fact, only sporadically gathered and consumed in northern Italy (the opposite of southern Italy [45]) and would certainly never represent “the” prototype within the wild vegetable group in Alpine areas. *Crumbotti* are instead still gathered, in areas close to houses/villages, during the spring by almost every woman in the study area, and they are either consumed in salads, boiled, or used as filling in home-made festive ravioli.

*Cicerbita alpina*, a relatively rare wild vegetable species which grows high in the mountains, is similarly popular in another area of northern Italy (the Alpine north-east) and has indeed been the subject of increasingly threatening overexploitation in recent years (Pieroni, personal observation). The fact that *Cicerbita alpina* grows in pristine high areas and is difficult to find, together with its delicate taste, may explain its increasing culinary prestige. Conversely, *crumbotti* seem to be more widely appreciated since they grow in anthropogenic environments close to the centres of inhabited villages (while the surrounding woodlands and pastures have been fully abandoned, see below) and are therefore easy to find, and they are certainly also considered part of the local culinary identity because of their appreciated non-bitter taste.

As in other areas of Northern and Central Italy, primrose (*Primula vulgaris*) and mallow (*Malva sylvestris*) teas remain very popular in the study area [e.g. 50–52].

### Temporal dynamics of plant and mushroom knowledge

It is crucial to note that the overlap between the taxa in the tables (Tables 1 and 2) is extremely limited, and only three genera (*Mentha*, *Rosa*, and *Sedum*; in bold in Table 2) were recorded in both the previous study and in the current one. This may be due to the fact that these genera were and still are commonly used or quoted: apart from mint teas (ubiquitously used across Europe), rose petal-based preparations (as an external remedy) and *Sedum* leaves also seem to be part of local food knowledge. The extremely clear overlapping of the two data sets suggests that the LEK linked to herbal teas and remedies has been lost, however, and “new” uses have emerged.

We also collected information about foraged items which are only remembered and no longer actively practiced: this was especially the case for on the spot “snacking” of clove flowers (*Trifolium* spp.), sorrel stems (*Rumex acetosella*), whitebeam (*Sorbus aria*), hawthorn berries (*Crataegus* spp.), and Cornelian cherries (*Cornus mas*), as well as for the gathering of wild apples (*Malus sylvestris*) and wild pears (*Pyrus pyraster*) for the preparation of home-made ciders and vinegars.

The gathering of wild tree fruits was alive as a practice while agro-silvo-pastoral activities survived, as this knowledge is based on daily interactions with woodlands and pastures as part of the local management practices of common land (*comunaglie*) widespread in the Ligurian Apennines. The gathering and use of some wild herbal remedies have been completely abandoned today. Three herbs seem to have been significant for the local collective memory: *Arnica montana*, *Polygala vulgaris*, and *Stachys annua* (locally known as *gerba*). Locals remember that these wild plants were gathered in large quantities in the past decades ago, both for domestic herbal uses and especially for selling to intermediaries and herbalists from neighbouring cities. The decline in herbal ingredients is surely linked not only to the dramatic abandonment of many natural *endroit* where community life took place until the 1970s, but also to the very common processes that take place in all rural areas with the arrival of urbanisation and the more pervasive coexistence with pharmaceuticals.

On the other hand, in Table 1 we indicated, with “N”, the uses that the study participants perceived as “new”, having been recently introduced (during their lifetime), and a few of these cases deserve to be discussed.

According to our informants, the aerial parts of ramson (*Allium ursinum*) were never gathered/used in the past in the study area, but city visitors from Genoa introduced a few years ago the custom (still sporadic) of collecting and using this plant as a substitute for garlic (and basil leaves) in one of the most iconic Ligurian culinary preparations: pesto (a sauce said to have originated in Genoa and/or the Ligurian and Nice coast, consisting of crushed garlic, Mediterranean pine nuts, coarse salt, basil leaves, and ewe cheese, all blended together with extra virgin olive oil), which is mainly used for dressing pasta noodles. The introduction “from the city” of the use of *Allium ursinum*, which grows widely in beech woodlands in spring in the upper Borbera Valley, testifies to a phenomenon that is well known in southern Europe, where this plant was introduced a few decades ago as a panacea, and also possibly thanks to the terrific popularity it achieved when the books by Austrian herbalist Maria Treben (1907–1991), especially “Health Through God's Pharmacy” [53], which strongly advocated its use, were translated, and became best-sellers in many countries.

**Fig. 5:**
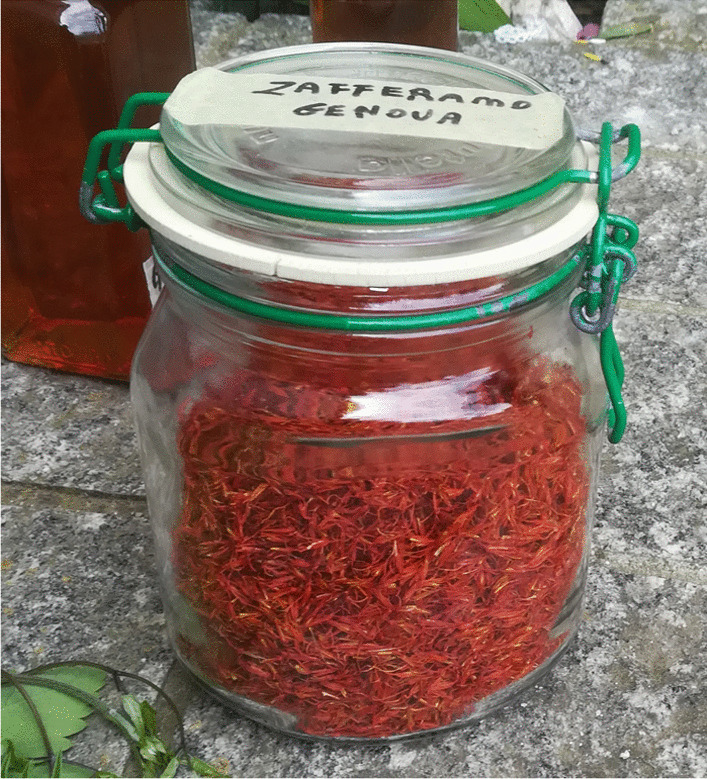
Dried home-gardened safflower (“zafferano Genova”) (Photograph: Andrea Pieroni)

Another interesting case is safflower (*Carthamus tinctorius*, Fig. 5), whose cultivation and use as a substitute for the more expensive saffron in the typical north Italian risotto were also reported to have come from Genoa, where the harbour-centred labour possibly enabled locals to encounter and become familiar with this new “spice” (mainly coming from and used in Turkey and the Middle East). Genoa and the coastal Tyrrhenian side of Liguria were again considered by the study participants as areas from which another “new” plant ingredient was introduced: the peel of bitter oranges (*Citrus x aurantium*), widely grown along the Ligurian coast and increasingly used in the study area for preparing home-made digestive wines.

Again, frequent contact with “city visitors” from Genoa was considered to be responsible for the emerging practice of foraging and cooking the mushroom *Craterellus cornucopioides*, which was previously never gathered within the study area.

The huge discrepancies between the data sets we collected and that of the study conducted four decades ago (Tables 1 vs. 2) could also possibly be attributed to different methodological frameworks used for eliciting data, and especially the sampling (the criteria according to which study participants were selected). Unfortunately, no information was provided about the sampling methods adopted during the study conducted in the late 1970s.

### Landscape transformations

**Fig. 6:**
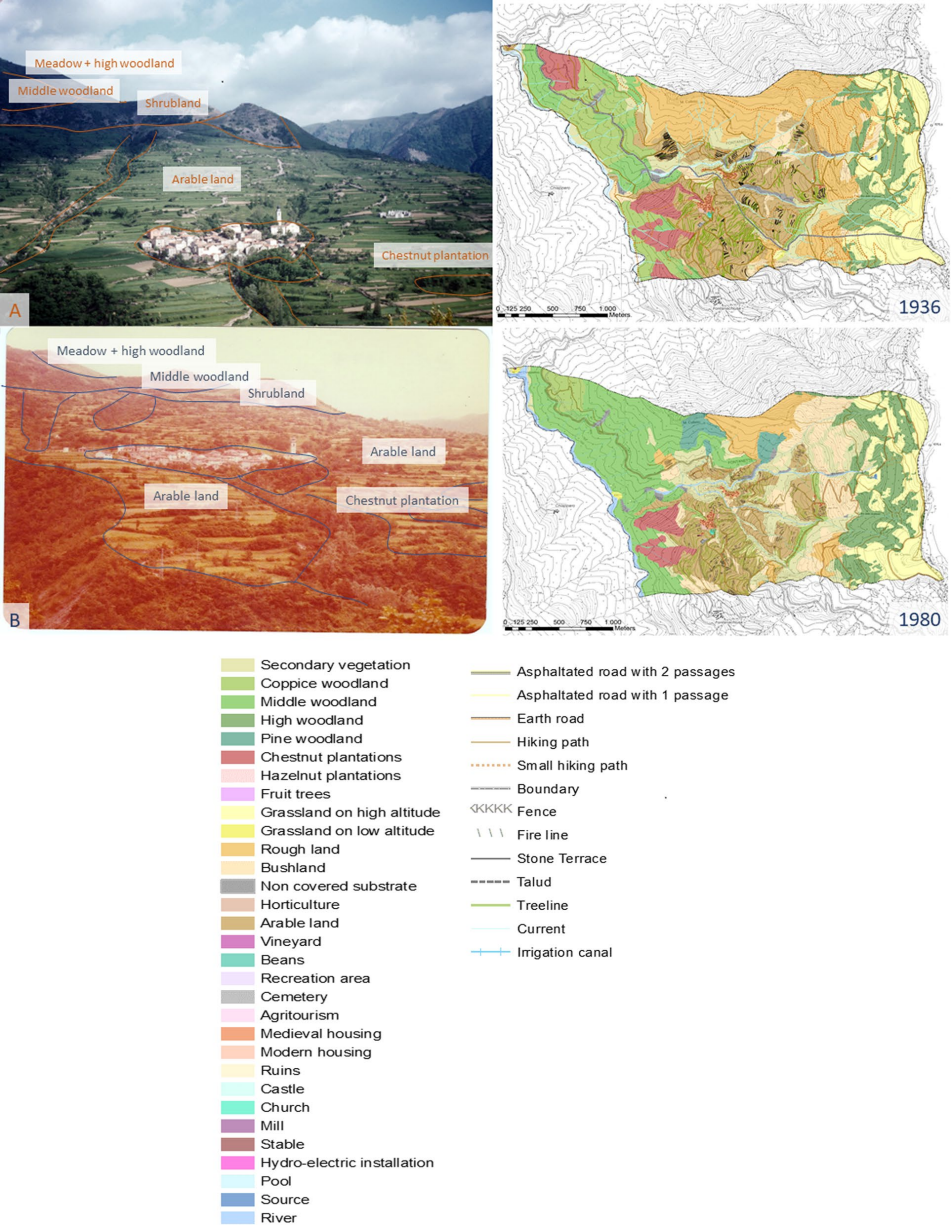
Carrega Ligure (**a**) in 1950 (Photograph: William John Crosetti) and (**b**) in 1977 (Photograph: Richard John Crosetti)

**Fig. 7:**
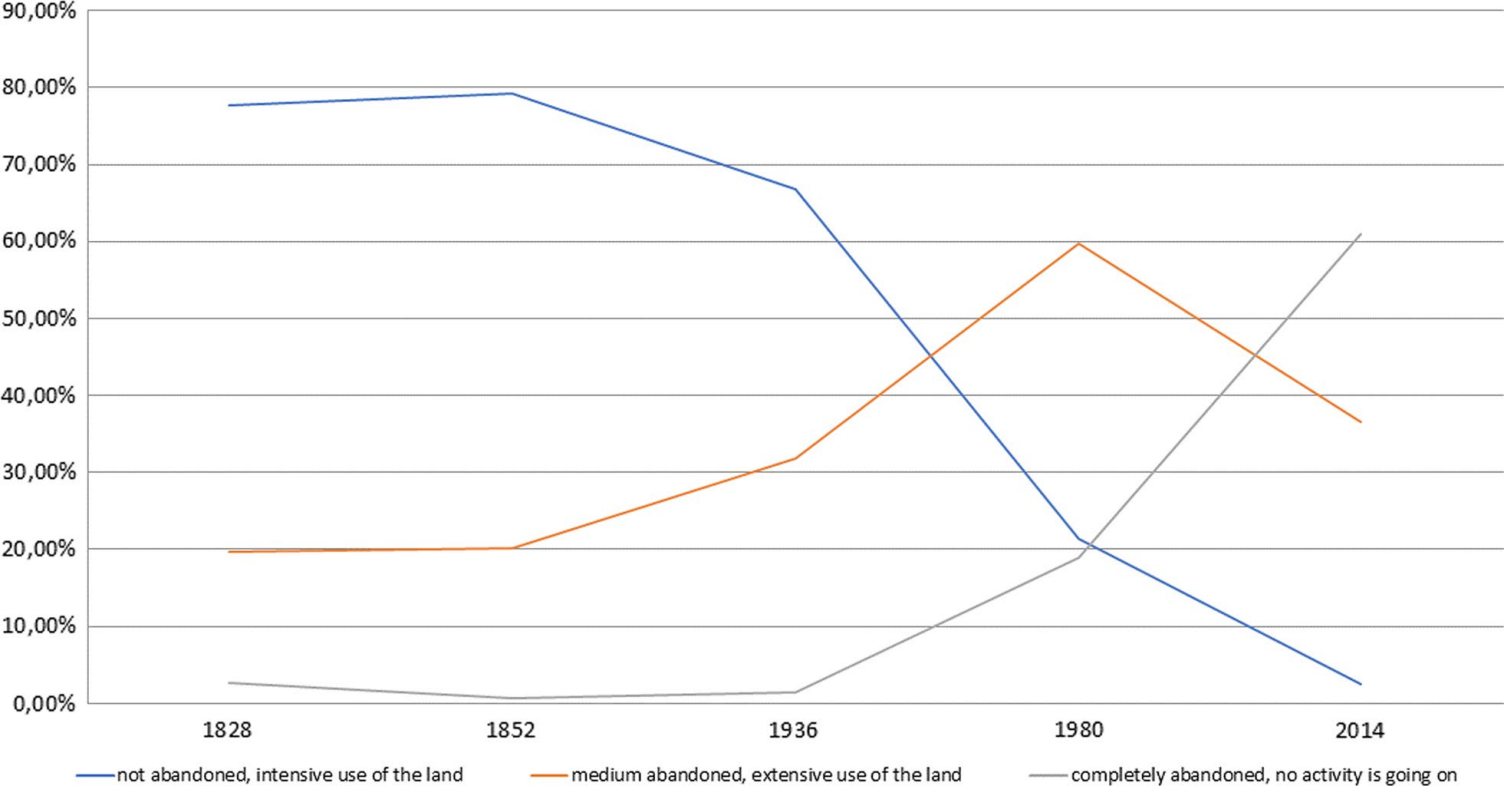
Evolution of landscape abandonment in Carrega Ligure during the last century

**Fig. 8:**
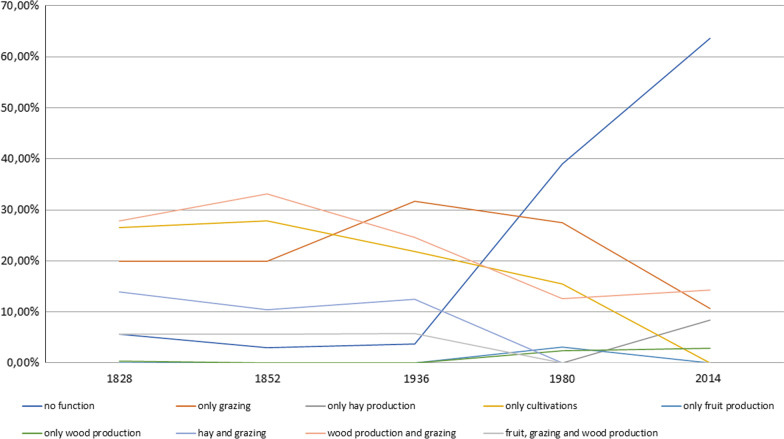
Landscape function/use in Carrega Ligure during the last century

As illustrated in Fig. 6, the current landscape of Carrega Ligure, located in the upper part of the Borbera Valley, is mainly characterised by woodlands, giving way to grasslands and shrublands on the top of Mount Carmo and the southern slope of Mount Colletto, respectively. The first impression of this type of landscape is its apparent “natural” value, but when we take a more in-depth look at the vegetative land cover, other land cover types and uses can be observed, and traces of past landscapes are revealed. In this section, a history of the landscape changes is described chronologically and illustrated in Figs. 7 and 8.

In the first half of the nineteenth century, the upper part of Borbera valley was dominated by an intensively (77.61%) organised agro-silvo-pastoral system. This historical rural system was based on five different functions: the grazing of cattle (sheep, cows, and goats); the production of hay; the cultivation of wheat, potatoes, vegetables, and corn, among other things; the production of fruit such as chestnuts, hazelnuts, peaches, and so on; and wood production. The land cover provided all these functions, as single or multiple uses. This intensive character was mostly related to the presence of cultivated land (31.83% in 1828) but also coppice woodlands (of both *Quercus cerris* and *Fagus sylvatica*) that were used for both wood production and grazing. The grasslands on the top of Mount Carmo were used for both hay production and cattle grazing (14.68%). Mount Colletto on the other hand was characterised by a less productive land cover, being shrubland, and was only used for the grazing of small livestock such as goats and sheep, and therefore the only land cover registered as extensive. Those grasslands and shrublands were part of historical common lands, managed by the inhabitants of Carrega and Connio through grazing rights. The cultivated lands close to the settlements were organised privately [54].

A decrease in intensity level, from 66.74% to 21.31%, was detected during the first half of the twentieth century. Cultivated lands, located on arable land in the surroundings of the settlements, remained rather stable and continued being used intensively. However, the grasslands lost the added function of hay production, concentrating only on cattle grazing. Grasslands were not the only land cover that changed. The change in land use was also related to a large invasion of secondary vegetation and mixed woodland, especially in less “interesting” and peripherical areas such as the former shrubland of Mount Colletto. Those steep and unforgiving areas were the first to be completely abandoned. Chestnut woodlands, located in the surroundings of the settlements, were almost completely abandoned from 1980 onwards, but retained the activity of fruit production, even if there was less demand. Coppice woodlands, in contrast, maintained the function of wood production, but, there again, in a more extensive way. The general process of extensification and simplification of land use throughout the twentieth century was mostly related to the shift from multiple to single use of the land cover. Some combinations of functions completely disappeared, especially the combination of hay production and grazing, and wood and fruit production alongside grazing activities. The combinations of different functions for a single land cover largely disappeared and a monofunctional organisation of the landscape emerged. These extensification and simplification processes were first noted in peripheral areas (outfield), and later moved towards the infield and higher meadows, with relevant ecological effects, as documented in other valleys of the Ligurian Apennines and beyond (a recent contribution by Piana et al. [55]).

The landscape of the upper Borbera valley is nowadays almost completely abandoned, as at least 60% of the territory no longer has any function or use. Those areas are recognisable by the dominant presence of invasive secondary vegetation and mixed woodland, which do not have any active function for the livelihood of local inhabitants. Approximately 6.5% of the landscape is used in an extensive way, meaning meadows and grasslands for hay production, woodlands for wood production, and high coppices, the last intensively used areas, for wood production and grazing activities.

This large extensification and even abandonment process is directly related to the high population pressure at the end of the nineteenth century. The historical agro-silvo-pastoral system which produced a highly intensively used landscape was no longer sufficient to feed the growing population of the nineteenth and early twentieth centuries.

Nonetheless, the upper Borbera Valley still offers extensive land uses such as cattle grazing on hillside pastureland in combination with wood production in high beech woodland, resulting, mainly, from previous wooded meadow pastures. The disappearance of the agro-silvo-pastoral system and the subsequently fast and drastic change of land use had a large impact on the land cover and on the ethnobotanical heritage of the landscape. So, apart from the disappearance of ethnobotanical knowledge due to demographic decline, a large number of local species related to the historical rural system are no longer found in the Apennine landscape.

## Discussion

Mountain and rural areas across Europe are facing depopulation and impoverishment, indicating a difficult future [7–9]. Local, national, and international institutions are designing and implementing strategies to counter this negative trend [55–60]. Particular attention is given to topics such as the physical and digital divide and funding the construction of mobility and information infrastructures. Alongside this, new initiatives have been developed in order to boost the economic attractiveness of these areas, supporting the establishment of new firms through tax relief and public investment. Tourism in particular is repeatedly referred to as a crucial rural development strategy because of its ability to create new, and reconnect former, rural peripheries to urban centres [61, 62]. In this context, tangible and, specifically, intangible heritage is perceived and employed as competitive assets for strengthening local attractiveness and creating initiatives concerning the reactivation of traditional knowledge, which often entails forms of heritage commodification [63]. This appears to be the context within which the case study of Carrega Ligure should be considered.

The theory of situationality recognises a strong link between the knowledge of a community, its activities and the surrounding environment [58]. Specifically, any change of knowledge within a community is the result of a combination of local socio-economic, demographic, and environmental changes. This assumption is useful for assessing the transformation in Carrega Ligure and opening the black box of the socio-economic impact of modernisation [64].

**Fig. 9:**
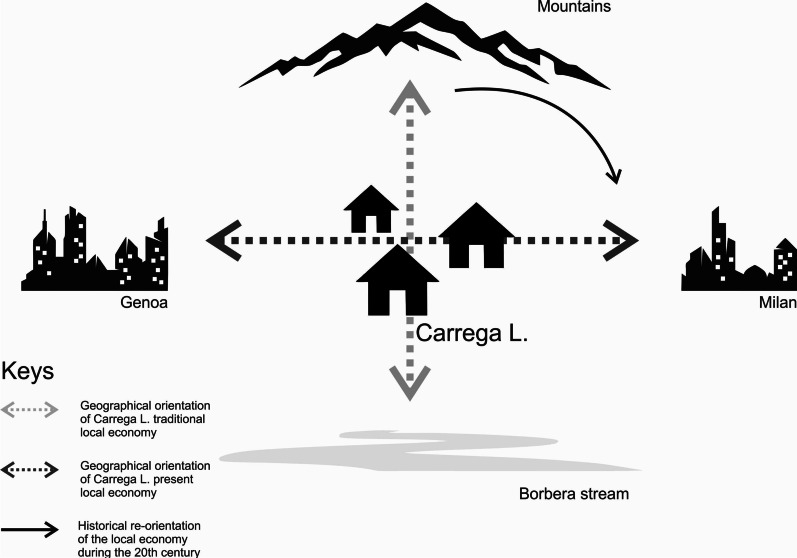
Changes in situational polarity of the economy and society of Carrega Ligure during the twentieth century (Figure: Michele F. Fontefrancesco)

Martini [33] surveyed the knowledge of older inhabitants of Carrega Ligure. These people had lived during their childhood and part of their adulthood within a social context based on traditional multifunctional agriculture, primarily aimed at self-subsistence [65]. The 15 taxa listed in the study delineate the borders of the human geography of the community that stretched from the bottom of the valley and the Borbera River to high pastures in the mountains. The space to which the LEK referred coincided with the space of economic activities. Ideally, it developed vertically and used wild botanical resources that grew in high pastures, in the interstitial space along the side of cultivated fields and gardens, or in the woods (Fig. 9). In this context, there was little economic and cultural integration with the world outside the village, which was limited to trade and migration.

After WWII, this socio-economic order entered into an irreversible crisis, when rapid industrialisation and economic growth accelerated the migration of members of the community to industrial centres mostly located within a 100 km distance [37]. This triggered a demographic decline that, in two decades, led to the closure of the local elementary school and the erosion of the network of local businesses. Currently, less than 40 people are permanent residents in the municipality of Carrega. However, the limited distance involved in the post-WWII migration allowed emigrants to easily return to their native community during the year (i.e. every weekend) and not only during summer holidays. The growth in such forms of commuting generated a stronger sociocultural connection between Carrega Ligure and large cities, such as Genoa and Milan, as well as local industrial centres, including Arquata, Serravalle Scrivia, and Novi Ligure. This connection was also reinforced by the erosion of the local network of public services that forced the inhabitants of Carrega Ligure to move to other cities to attend schools or have access to banks, churches, post offices, shops, and grocery stores. Accordingly, the economic and human geography of Carrega Ligure changed its spatial polarisation, abandoning both the valley and high pastures. It developed horizontally, along the road that passes through the village and connects it to other towns and cities. As shown in Fig. 7, this new polarisation coincided with the abandonment of terraces and the marginalisation of agriculture and cattle breeding as a primary source of income for local families, which in turn fostered the rewilding of the valley. This socio-economic integration has an impact on the very structure of community knowledge. LEK concerning taxa obtained from spaces no longer frequented on a daily basis was eroded, and, as reported in Table 1, new knowledge has been integrated into the community following interactions with people who come to Carrega Ligure from cities for work or tourism (for example, this is the situation for the LEK related to mushrooms, such as *Craterellus cornucopioides*, transmitted to locals by Genovese mushroom hunters who visit the woods of the municipality every autumn), or through knowledge acquired from mass media and cultural sources (for instance, the gathering of *Allium ursinum*, the cultivation of *Carthamus tinctorius,* and the use of *Citrus x aurantium* peels to make an aromatised wine).

The diachronic loss of LEK in the past half century can be understood, first of all, in light of the abandonment and transformation of ecological spaces, such as those related to terracing agriculture or high-altitude pastures. However, it is also a sign of the deeper integration of the community within the national cultural and economic system, as well as the strengthening of national public services. This is relevant in explaining the marginalisation of LEK linked to the use of medicinal plants that was still common before the institution of the Italian national health service in 1978 [66]. With the expansion of the health service from the 1980s onwards, and better access to modern drugs, including in Carrega Ligure, people abandoned traditional remedies in favour of the new ones, leading to the erosion of botanical knowledge and practices embedded in LEK.

People in Carrega Ligure recognise the ongoing knowledge loss and transformation, identifying one of the main drivers behind these trends in the socio-economic transformation of the community. In response, they have taken various actions aimed at recovering and preserving LEK and the underlying aspects of local food and ethnobotanical knowledge. These range from the establishment of the natural park to the renovation and reactivation of old watermills and kilns. As in other cases of folk revivals in the mountain areas of Northern Italy [67–71], the strategies undertaken in the study area have also been aimed at preserving local resources in the face of their erosion. However, while folk revival has mainly considered the preservation of traits of local intangible heritage [72, 73], strong emphasis is given in the case of Carrega Ligure to the importance of the preservation and promotion of the environmental heritage. This process suggests a change in the community’s relationship with the mountain landscape. While the post-war period was marked by the abandonment of this territory and the de-functionalisation and loss of the related LEK, the recent past indicates an incipient return to these places and to this knowledge. This is a re-appropriation of the mountain space through a reconstitution of the collective memory and knowledge, which could in turn foster the recovery and actualisation of traditional food and ethnobotanical heritage associated with these places. This dynamic, however, is not linked to an attempt to return to a traditional subsistence strategy. Rather, it is part of the development of tourism in the area and the valorisation of local productions. The case of Carrega Ligure thus follows a broader trajectory shared by other villages across the Apennines and the Alps, where tourism is embraced by local communities as the only viable path for development [71–76]. In an attempt to compete more strongly within the tourist market, traditional knowledge as well as local heritage is objectified and essentialised [77] in order to adapt to market demand. This in turn brings the risk of commodifying heritage [27] and making tourism a strategy that has few benefits in terms of improving the cultural, environmental, and social sustainability of the local foodscape and associated actors. The centrality given to tourism in Carrega Ligure, therefore, could also enable these scenarios. Thus, in this context, the current reactivation of traditional knowledge should not be considered as an indication of a possible return to the past geographical polarisation of the local economy. Rather, it confirms its horizontal disposition and suggests an intensification of the role played by cities in shaping the life of the community.

## Conclusions

This research analysed the diachronic transformation of LEK in the community of Carrega Ligure, in the Piedmontese Ligurian Apennines, in NW Italy. It compared the results found by Enrico Martini [33] in the municipality for the period 1976–1978 with the data collected in 2021 by the authors. The analysis interpreted the data from a situationist perspective, connecting the change in LEK to the socio-economic changes experienced by the community.

Since the end of WWII, Carrega Ligure has experienced a socio-economic structural change marked by the economic integration of the community in the national market that ended the previous system of mountain subsistence farming. These changes led to a transformation in the use of environmental resources that is echoed in the local LEK.

Knowledge loss affects those aspects linked to spaces no longer frequented or exploited by the community. In the last decade, however, the community began a process of rediscovery and promoting LEK. This revival is linked to the growing interest in tourism, seen as a promising avenue for economic development in this marginalised community. The sustainability of this process, from a socio-economic point of view, is linked to the ability of the community to reactivate LEK so as to meet the expectations of tourists.

LEK is playing a role in defining Carrega Ligure as a tourist destination, but the potentially crucial role of tourism in the renaissance of food and ethnobotanical knowledge raises serious questions about the sustainability of this process. While the rediscovery of these resources would help to strengthen the social cohesion of a fragile community as an element of collective identity, tourism risks bending the specificities of local culture to the exogenous expectations of tourists, furthering the sociocultural fragility of the community. To counter this possible degeneration, tourism should not be the only option available for a community to imagine its future, and public and private intervention is needed to pluralise the local economy in terms of sectors of development, and thus to support the emergence of new enterprises. Unfortunately, this is not what is happening, leaving tourism the only beacon on the horizon.

This research confirms the limits of diachronic analysis based on the comparison of historical data collected in previous decades, when ethnobotany did not yet have a clear methodological framework and the bibliographic sources do not explain the methodology used in detail. Under these conditions, the comparison only maintains a qualitative meaningfulness that needs the support of other, historical, and ethnographic sources, to fully shed light on the transformations that occurred in a community.

## Data Availability

The original data set for the work published in 1977 is available via the authors’ address and in Table 2. Data set of the work conducted in 2021 is presented in Table 1.
